# New Species of *Neocosmospora* (Ascomycota) from China as Evidenced by Morphological and Molecular Data

**DOI:** 10.3390/life13071515

**Published:** 2023-07-06

**Authors:** Zhao-Qing Zeng, Wen-Ying Zhuang

**Affiliations:** State Key Laboratory of Mycology, Institute of Microbiology, Chinese Academy of Sciences, Beijing 100101, China; zhuangwy@im.ac.cn

**Keywords:** Hypocreales, morphology, Nectriaceae, phylogeny, taxonomy

## Abstract

Species of *Neocosmospora* are commonly found in soil, plant debris, and living woody or herbaceous substrates and occasionally found in water and air. Some species are reported as saprobes, endophytes, opportunistic pathogens of plants and animals, or producers of bioactive natural products, cytotoxic compounds, and industrial enzymes. To reveal the species diversity of *Neocosmospora*, specimens from different provinces of China were investigated. Five new species, *Neocosmospora anhuiensis*, *N. aurantia*, *N. dimorpha*, *N. galbana*, and *N. maoershanica*, were introduced based on morphological characteristics and DNA sequence analyses of combined calmodulin (CAM), the internal transcribed spacer (ITS), the second largest subunit of RNA polymerase II (RPB2), and the translation elongation factor 1-α (TEF1) regions. Differences between these new species and their close relatives are compared in detail.

## 1. Introduction

*Neocosmospora* E.F. Sm., typified by *N. vasinfecta* E.F. Sm., is characterized by superficial, globose to pyriform perithecia that are yellow, orange-brown, or red, KOH+, LA+, and coarsely warted; cylindrical to narrowly clavate asci containing eight ascospores; globose to ellipsoidal, 0–1-septate ascospores; oval, ellipsoidal, or subcylindrical, 0–1-septate microconidia; and subcylindrical, multiseptate macroconidia with the tips cell slightly hooked [1,2]. About 129 epithets have been listed under generic names (www.indexfungorum.org), among which 102 species are commonly accepted [2,3,4,5,6,7,8,9]. They are mainly distributed in tropical and subtropical regions and are commonly found in soil, plant debris, and living woody or herbaceous materials, occasionally in air and water, and rarely in human tissues [2,9].

*Neocosmospora* species are economically important in industrial, agriculture, and human health fields [10]. For example, *N. solani* (Mart.) L. Lombard & Crous can produce bioactive compounds and various enzymes with industrial utilization including hydrolases and laccases [11,12,13]. On the other hand, many of them are opportunistic phytopathogens that cause cankers, stem and root rot, and cane blight of numerous plants [5,8,10,14,15,16,17,18,19,20,21,22,23,24,25], while a few species were reported as human pathogens [10]. Enhancing and updating our knowledge of *Neocosmospora* will provide useful information about the maintainable utilization of natural resources and protection against harmful species.

In connection with our recent research on the Chinese fungus flora, five undescribed species of *Neocosmospora* were encountered based on their perithecial gross morphology, anatomy, and culture characteristics. Their taxonomic placements were confirmed by DNA sequence analyses of combined CAM, ITS, RPB2, and TEF1 loci. Comparisons between the new species and their close relatives were made.

## 2. Materials and Methods

The collections examined were found on wood substrates from Anhui, Hubei, Hunan Provinces, and the Guangxi Zhuang Autonomous Region of China and are deposited in Herbarium Mycologicum Academiae Sinicae (HMAS) in China. The methods used by Luo and Zhuang [26] were generally followed for morphological observations. Perithecial wall reactions were tested in 3% potassium hydroxide (KOH) and 100% lactic acid (LA). To observe the microscopic characteristics of the perithecial wall, sections were made with a freezing microtome YD-1508-III (Jinhua, China) at a thickness of 6–8 μm. Macroscopic photographs were taken with the digital camera Leica DFC450 (Wetzlar, Germany) attached to the stereomicroscope Leica M125 (Milton Keynes, UK), and microscopic features were recorded using the digital camera Zeiss AxioCam MRc 5 (Jena, Germany) attached to the microscope Zeiss Axio Imager A2 (Göttingen, Germany). Cultures were obtained from fresh ascomata using single ascospore isolation. The colony morphology was observed by growing on potato dextrose agar (PDA) and synthetic nutrient-poor agar (SNA) [27] at 25 °C in an incubator with alternating periods of light and darkness (12 h/12 h). Growth rates of the colony were measured after 7 d.

The genomic DNA was extracted from fresh mycelium following the methods of Wang and Zhuang [28]. Sequences of CAM, ITS, RPB2, and TEF1 were amplified using primer pairs CL1/CL2A [29], ITS5/ITS4 [30], RPB2-5f/RPB2-7cR [31], and EF1/EF2 [32], respectively. Sequences were assembled, aligned, and manually edited by using BioEdit 7.0.5 [33] and altered to NEXUS files by using ClustalX 1.8 [34].

To determine the phylogenetic positions of the Chinese collections, sequences of CAM, ITS, RPB2, and TEF1 were combined and analyzed with Bayesian inference (BI) and maximum likelihood (ML) analyses. The BI analysis was executed by using MrBayes 3.1.2 [35] using a Markov chain Monte Carlo algorithm. The ML analysis was conducted by using IQ-Tree 1.6.12 [36] using the best model for each locus chosen via ModelFinder [37]. Nucleotide substitution models were determined by using MrModeltest 2.3 [38]. Four Markov chains were run simultaneously for 1,000,000 generations with the trees sampled every 100 generations. The Bayesian inference posterior probability (BIPP) was determined from the remaining trees. Trees were examined in TreeView 1.6.6 [39], with BIPP greater than 0.9 and maximum likelihood bootstrap (MLBP) greater than 70% showing at the nodes.

## 3. Results

### 3.1. Phylogenetic Analyses

**Figure 1:**
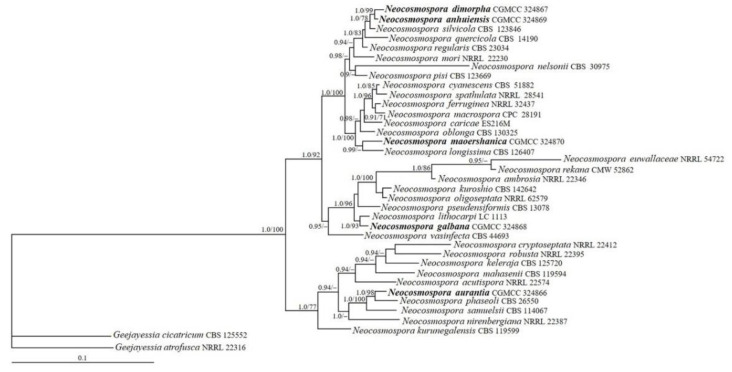
The Bayesian inference tree created based on CAM, ITS, RPB2, and TEF1 sequences of *Neocosmospora* species. BIPP (**left**) above 0.9 and MLBP (**right**) above 70% are indicated at nodes.

The sequences of CAM, ITS, RPB2, and TEF1 from 35 representative *Neocosmospora* species that are closely related to the five new taxa based on BLAST searches were analyzed (Table 1). The resulting BI tree is displayed in Figure 1. The topology of the ML tree was similar to that of the BI tree. The strains CGMCC 3.24866, 3.24867, 3.24868, 3.24869, and 3.24870 were grouped with other members of *Neocosmospora* receiving high statistical support values (BIPP/MLBP = 1.0/100%). The strain CGMCC 3.24867 and 3.24869 were closely related (BIPP/MLBP = 1.0/99%), which were further grouped with *N. silvicola* Sand.-Den. & Crous (BIBP/MLBP = 1.0/78%). The strain CGMCC 3.24870 was clustered with *N. longissima* Sand.-Den. & Crous (BIBP = 0.99), isolate CGMCC 3.24868 was related to *N. lithocarpi* M.M. Wang & L. Cai (BIBP/MLBP = 1.0/93%), and the remaining strain CGMCC 3.24866 was associated with *N. phaseoli* (Burkh.) L. Lombard & Crous (BIBP/MLBP = 1.0/98%).

### 3.2. Taxonomy

**Figure 2:**
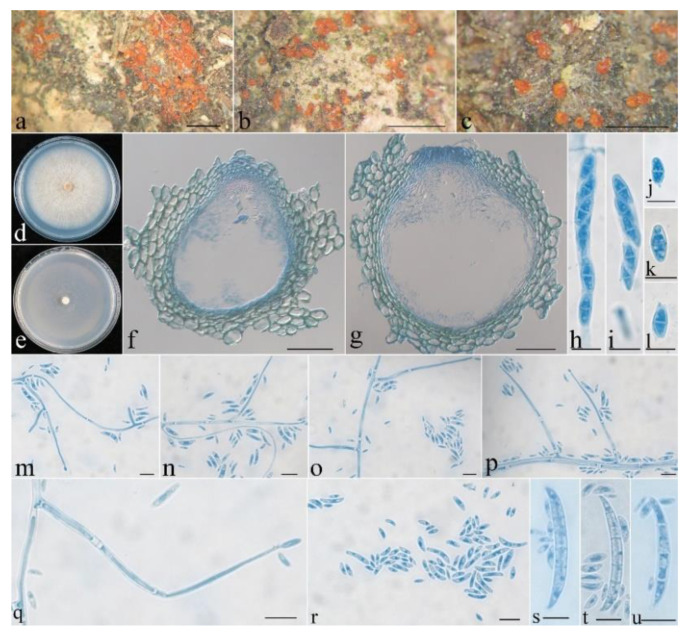
*Neocosmospora anhuiensis* (HMAS 255836). (**a**–**c**) Perithecia on rotten twigs. (**d**,**e**) Colonies after 1 wk at 25 °C ((**d**) on PDA, (**e**) on SNA). (**f**,**g**) Longitudinal section through perithecium. (**h**,**i**) Ascus with ascospores. (**j**–**l**) Ascospore. (**m**–**q**) Conidiophores and microconidia. (**r**) Microconidia. (**s**–**u**) Macroconidia and microconidia. Scale bars: (**a**–**c**) = 1 mm, (**f**,**g**) = 50 μm, (**h**–**u**) = 10 μm.

***Neocosmospora anhuiensis*** Z.Q. Zeng & W.Y. Zhuang, sp. nov. Figure 2.

Fungal Names: FN 571307.

Etymology: The specific epithet refers to the type locality.

Typification: CHINA. Anhui Province, Huangshan, Yungu Temple, on rotten twigs, 22 June 2019, Z.Q. Zeng & H.D. Zheng 12364 (holotype HMAS 255836, ex-type culture CGMCC 3.24869).

Mycelium was visible neither around ascomata nor on natural substrates. The features of the ascomata were as follows: perithecial, superficial, solitary to gregarious, non-stromatic, subglobose to globose, or pyriform; showing lateral collapse upon drying; orange-red, turning dark red in 3% KOH, becoming light yellow in 100% LA; and 196–245 × 186–255 μm. The features of the perithecial surface were as follows: coarsely warted, of textura angularis to textura globosa, warts 15–40 µm high, cells 10–25 × 8–18 µm, and walls 0.8–1 µm thick. The perithecial wall of two layers was 28–43 µm thick; the outer layer of textura angularis was 23–33 μm thick, the cells were 10–18 × 6–13 μm, the walls were 1–1.2 μm thick; and the inner layer of textura prismatica was 5–10 μm thick, the cells were 8–15 × 2–3 μm, the walls were 0.8–1 μm thick. Asci were cylindrical to clavate, with a round and simple apex, 8-spored and 48–70 × 5–9 µm. Ascospores were ellipsoidal, 1-septate, hyaline, smooth-walled, irregularly biseriate, and 8–15 × 3–5 µm.

On PDA, the colony was 77 mm in diam. after 7 d at 25 °C, and the surface was cottony, with a dense, whitish aerial mycelium forming pale yellow pigments. On SNA, the colony was 70 mm in diam. after 7 d at 25 °C, and the surface was velvety, with a sparse, whitish aerial mycelium. Conidiophores ranged from unbranched to simply branched, with indefinite length. Microconidia ranged from ellipsoidal to rod-shaped and straight to slightly curved and were hyaline, smooth-walled, 0–1-septate, and 4.5–15 × 1.5–2.5 μm. Macroconidia were falcate, 2–6-septate, hyaline, smooth-walled, and 25–58 × 3.5–5 μm. Chlamydospores were not observed.

Notes: Morphologically, *N. anhuiensis* most resembles *N. pseudensiformis* Samuels in having subglobose, coarsely warted perithecia, which show lateral collapse upon drying, with clavate asci, ellipsoidal microconidia, and falcate macroconidia and lack the capacity of producing chlamydospores in culture [40]. However, the latter has larger asci (60–100 × 7–11.5 μm); broadly ellipsoidal, pale yellow-brown, and striate ascospores; a tan colony on PDA; and wider macroconidia (4.5–9 μm wide). Phylogenetically, they are remotely related (Figure 1).

**Figure 3:**
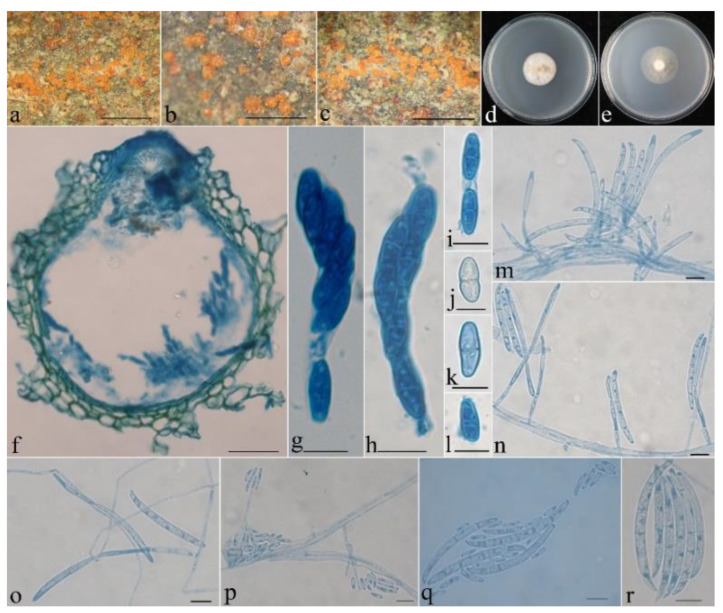
*Neocosmospora aurantia* (HMAS 290899). (**a**–**c**) Perithecia on rotten bark. (**d**,**e**) Colonies after 1 wk at 25 °C ((**d**) on PDA, (**e**) on SNA). (**f**) Longitudinal section through perithecium. (**g**,**h**) Ascus with ascospores. (**i**–**l**) Ascospore. (**m**–**o**) Conidiophores and macroconidia. (**p**) Conidiophores and microconidia. (**q**) Macroconidia and microconidia. (**r**) Macroconidia. Scale bars: (**a**–**c**) = 1 mm, (**f**) = 50 μm, (**g**–**r**) = 10 μm.

***Neocosmospora aurantia*** Z.Q. Zeng & W.Y. Zhuang, sp. nov. Figure 3.

Fungal Names: FN 571308.

Etymology: The specific epithet refers to the orange-colored perithecia.

Typification: CHINA. Hubei Province, Shennongjia forestry district, Muchengshaoqia, on rotten bark, 22 September 2014, Z.Q. Zeng, H.D. Zheng, W.T. Qin & K. Chen 10053 (holotype HMAS 290899, ex-type culture CGMCC 3.24866).

Mycelium was visible neither around ascomata nor on natural substrates. The features of the ascomata were as follows: perithecial, superficial, solitary to gregarious, non-stromatic or with a basal stroma, subglobose to globose, or pyriform; orange-yellow when fresh, yellow-orange when dry, turning dark red in 3% KOH, becoming light yellow in 100% LA; and 235–304 × 206–323 μm. The features of perithecial surface were as follows: slightly warted, of textura angularis to textura globosa, warts 15–63 µm high, cells 10–30 × 8–13 µm, and walls 0.8–1 µm thick. The perithecial wall of two layers was 18–30 µm thick; the outer layer of textura angularis to textura globosa was 13–23 μm thick, the cells were 10–25 × 5–22 μm, the walls were 0.8–1 μm thick; and the inner layer of textura prismatica was 5–8 μm thick, the cells were 5–10 × 2–3 μm, the walls were 0.9–1.2 μm thick. Asci were cylindrical to clavate, with a round and simple apex, 8-spored and 43–75 × 5–12 µm. Ascospores were ellipsoidal to oblong, 1-septate, hyaline, smooth-walled, uniseriate or irregularly biseriate, and 10–16 × 4–5 μm.

On PDA, the colony was 28 mm in diam. after 7 d at 25 °C, and the surface was floccose, with a dense, whitish aerial mycelium forming pale brown pigments. On SNA, the colony was 33 mm in diam. after 7 d at 25 °C, and the surface was floccose, with a sparse, whitish aerial mycelium. Conidiophores were simply branched, 16–83 μm long, and 2–3 μm wide at the base. Microconidia ranged from ellipsoidal to rod-shaped and were hyaline, smooth-walled, 0(–1)-septate, and 4–15 × 1.5–2.5 μm. Macroconidia were mainly falcate, rarely cylindrical, slightly curved, (1–)3–4(–5)-septate, hyaline, smooth-walled, and 43–75 × 5–6 μm.

Notes: The species is morphologically most similar and phylogenetically related to *N. phaseoli* (BIPP/MLBP = 1.0/98%) (Figure 1) in having dense floccose, white aerial mycelium, sparsely branched conidiophores, ellipsoidal microconidia with 0(–1)-septate, and falcate macroconidia with 3–4-septate [41]. However, the latter differs in its shorter macroconidia (32–58 μm long), larger microconidia (13.5–32.5 × 3.5–6 μm), and the production of subglobose to ellipsoidal chlamydospores [41]. Moreover, the sequence comparisons display that there are 19 bp, 22 bp, 22 bp, and 6 bp differences in the regions of CAM, ITS, RPB2, and TEF1, respectively. They are not conspecific.

**Figure 4:**
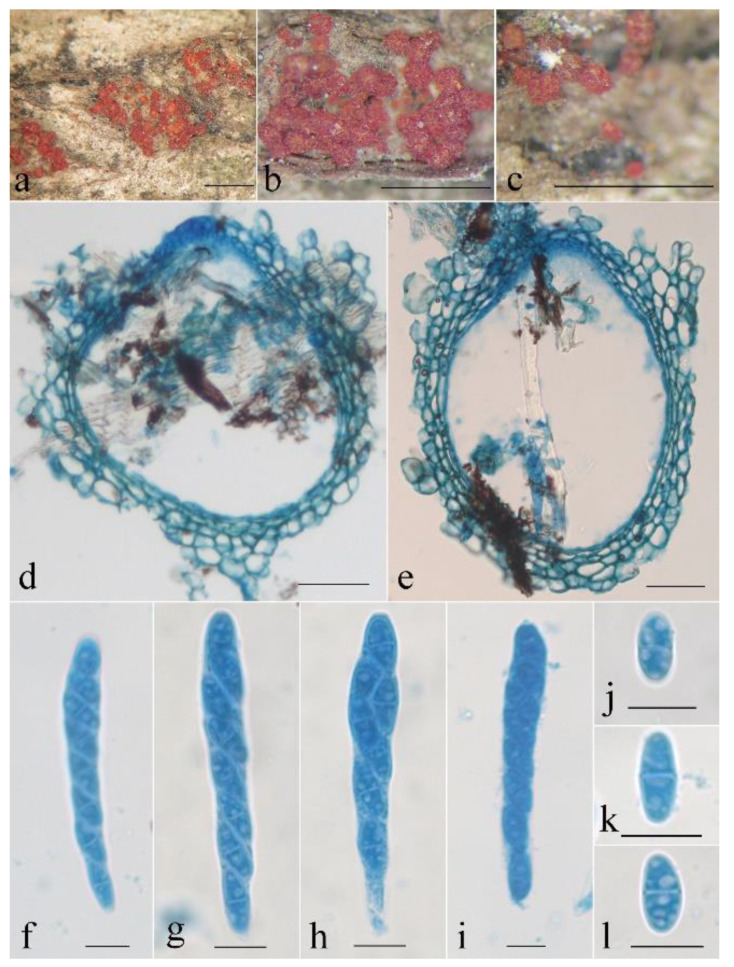
*Neocosmospora dimorpha* (HMAS 255837). (**a**–**c**) Perithecia on rotten twigs. (**d**,**e**) Longitudinal section through perithecium. (**f**–**i**) Ascus with ascospores. (**j**–**l**) Ascospore. Scale bars: (**a**–**c**) = 0.5 mm, (**d**,**e**) = 50 μm, (**f**–**l**) = 10 μm.

**Figure 5:**
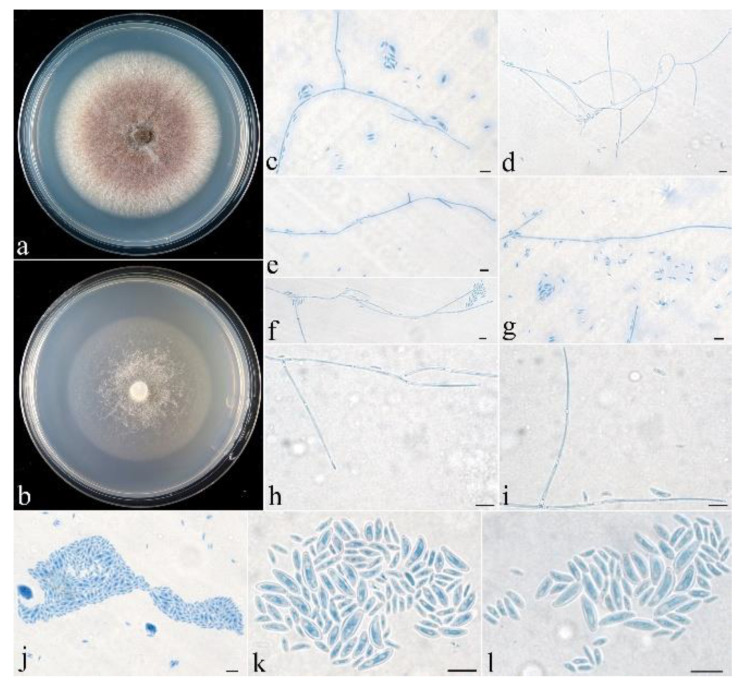
*Neocosmospora dimorpha* (CGMCC 3.24867). (**a**,**b**) Colonies after 1 wk at 25 °C ((**a**) on PDA, (**b**) on SNA). (**c**–**i**) Conidiophores and microconidia. (**j**–**l**) Microconidia. Scale bars: (**c**–**l**) = 10 μm.

***Neocosmospora dimorpha*** Z.Q. Zeng & W.Y. Zhuang, sp. nov. Figure 4 and Figure 5.

Fungal Names: FN 571309.

Etymology: The specific epithet refers to the presence of two types of microconidia.

Typification: CHINA. Hunan Province, Hengyang, Nanyue scenic spot, on rotten twigs, 21 October 2015, Z.Q. Zeng, X.C. Wang, K. Chen & Y.B. Zhang 10144 (holotype HMAS 255837, ex-type culture CGMCC 3.24867).

Mycelium was visible neither around ascomata nor on natural substrates. The features of the ascomata were as follows: perithecial, superficial, solitary to gregarious, non-stromatic, subglobose to globose, or pyriform; orange-red, turning dark red in 3% KOH, becoming light yellow in 100% LA; and 225–294 × 196–254 μm. The features of perithecial surface were as follows: slightly warted, of textura angularis to textura globosa, warts 15–45 µm high, cells 8–28 × 5–20 µm, and walls 0.8–1 µm thick. The perithecial wall of two layers was 15–40 µm thick; the outer layer of textura angularis to textura globosa was 10–30 μm thick, the cells were 5–15 × 4–10 μm, the walls were 1–1.2 μm thick; and the inner layer of textura prismatica was 5–10 μm thick, the cells were 8–14 × 2–3 μm, the walls were 0.8–1 μm thick. Asci were cylindrical to clavate, with a round and simple apex, 8-spored and 63–80 × 5.5–10 µm. Ascospores were ellipsoidal, 1-septate, hyaline, smooth-walled, uniseriate or irregularly biseriate, and 8–15 × 3.5–5 μm.

On PDA, the colony was 67 mm in diam. after 7 d at 25 °C, and the surface was cottony, with a dense, whitish aerial mycelium forming pale brown pigments. On SNA, the colony was 58 mm in diam. after 7 d at 25 °C, and the surface was floccose, with a sparse, whitish aerial mycelium. Conidiophores ranged from unbranched to simply branched, with indefinite length. Microconidia were ellipsoidal or rod-shaped: ellipsoidal microconidia were straight to slightly curved, unseptate, hyaline, smooth-walled, and 4–10.6 × 1.6–4.1 μm; rod-shaped microconidia were slightly curved, 0(–1)-septate, hyaline, smooth-walled, and 4–14.2 × 1.6–5.2 μm. Macroconidia and chlamydospores were not observed.

Notes: Amongst the existing species of *Neocosmospora*, *N. dimorpha* is morphologically similar and phylogenetically related to *N*. *anhuiensis* in having solitary to gregarious, non-stromatic, subglobose to globose, or pyriform perithecia; cylindrical to clavate asci; ellipsoidal ascospores; and an absence of chlamydospores. However, *N*. *anhuiensis* differs in having somewhat shorter asci (48–70 µm long), having faster colony growth rates on PDA and SNA (77 mm and 70 mm in diam.), producing a pale yellow pigment on PDA, and forming falcate, 2–6-septate macroconidia in culture. Additionally, there are 9 bp, 11 bp, and 3 bp differences in the CAM, ITS, and TEF1 regions, respectively, between the type strains (CGMCC 3.24867 and 3.24869).

**Figure 6:**
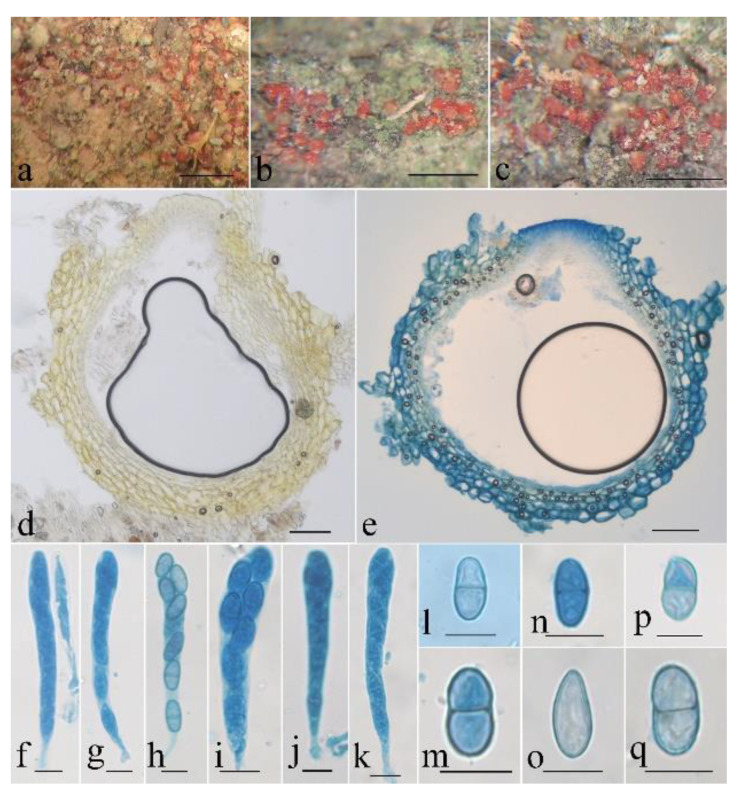
*Neocosmospora galbana* (HMAS 247874). (**a**–**c**) Perithecia on rotten bark. (**d**,**e**) Longitudinal section through perithecium. (**f**–**k**) Ascus with ascospores. (**l**–**q**) Ascospore. Scale bars: (**a**–**c**) = 1 mm, (**d**,**e**) = 50 μm, (**f**–**q**) = 10 μm.

**Figure 7:**
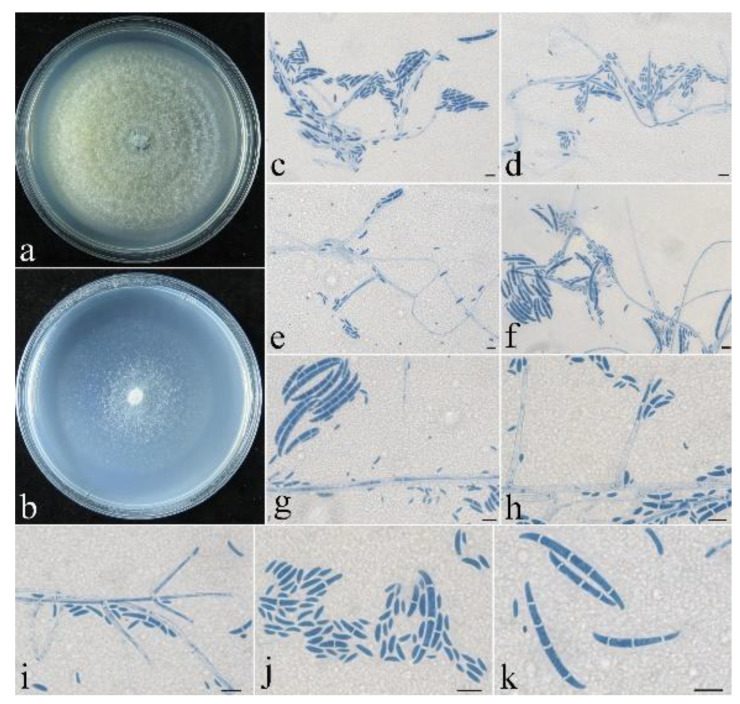
*Neocosmospora galbana* (CGMCC 3.24868). (**a**,**b**) Colonies after 1 wk at 25 °C ((**a**) on PDA, (**b**) on SNA). (**c**–**i**) Conidiophores, macroconidia and microconidia. (**j**,**k**) Macroconidia and microconidia. Scale bars: (**c**–**k**) = 10 μm.

***Neocosmospora galbana*** Z.Q. Zeng & W.Y. Zhuang, sp. nov. Figure 6 and Figure 7.

Fungal Names: FN 571310.

Etymology: The specific epithet refers to the greenish-yellow colony on PDA.

Typification: CHINA. Hubei Province, Shennongjia, Banqiao, on rotten bark, 20 September 2014, Z.Q. Zeng, H.D. Zheng, K. Chen & W.T. Qin 9942 (holotype HMAS 247874, ex-type culture CGMCC 3.24868).

Mycelium was visible neither around ascomata nor on natural substrates. The characteristics of the ascomata are as follows: perithecial, superficial, solitary to gregarious, non-stromatic or with a basal stroma, subglobose to globose, or pyriform; orange-red to brownish-red, turning dark red to violet in 3% KOH, becoming light yellow in 100% LA; and 225–304 × 216–333 μm. The features of perithecial surface were as follows: warted, of textura angularis to textura globosa, warts 12–63 µm high, cells 10–32 × 8–13 µm, and walls 0.8–1 µm thick. The perithecial wall of two layers was 25–45 µm thick; the outer layer of textura angularis to textura globosa was 10–37 μm thick, the cells were 5–15 × 4–8 μm, the walls were 1–1.2 μm thick; the inner layer of textura prismatica was 5–8 μm thick, the cells were 10–20 × 2–3 μm, the walls were 0.8–1 μm thick. Asci were cylindrical to clavate, with a round and simple apex, 8-spored and 63–88 × 7.5–12 μm. Ascospores were ellipsoidal, (0–)1-septate, hyaline, smooth-walled, uniseriate or irregularly biseriate, and 8–13 × 4–5.5 μm.

On PDA, the colony was 84 mm in diam. after 7 d at 25 °C, and the surface was cottony, with a dense, whitish aerial mycelium forming yellowish-green pigments. On SNA, the colony was 58 mm in diam. after 7 d at 25 °C, and the surface was floccose, with a sparse, whitish aerial mycelium. Conidiophores were acremonium- to verticillium-like, with a whorl of 2–4 phialides, and the phialides were subulate to cylindrical, 16–58 μm long, and 1.5–2 μm wide at the base. Microconidia were ellipsoidal to rod-shaped, 0(–1)-septate, hyaline, smooth-walled, and 4–8 × 1.5–2.5 μm. Macroconidia were falcate, 1–6-septate, hyaline, smooth-walled, and 13–73 × 2.5–5 μm.

Notes: Among the existing species of the genus, *N. galbana* is morphologically most related to *N. pseudensiformis* Samuels in having subglobose perithecia, clavate asci, acremonium- to verticillium-like conidiophores, and falcate macroconidia [40]. Nevertheless, the latter has pale yellow-brown, striate, and longer ascospores (10–16.2 μm long), a tan colony on PDA, and wider macroconidia (3.5–9 μm wide). Phylogenetically, *N. galbana* clustered with *N. lithocarpi* (BIBP/MLBP = 1.0/93%). The sequence comparison of the type cultures indicated that there are 3 bp, 16 bp, and 5 bp differences detected for ITS, RPB2, and TEF1 regions, respectively. Moreover, *N. lithocarpi*, only known for its asexual stage, differs in having a slower colony growth rate on PDA (57–59 mm in diam.), a greyish-orange pigment in culture, wider macroconidia (3.9–8.1 μm wide) with 5 septa, and larger microconidia (7–24 × 3.5–7 μm) and producing abundant chlamydospores [9].

**Figure 8:**
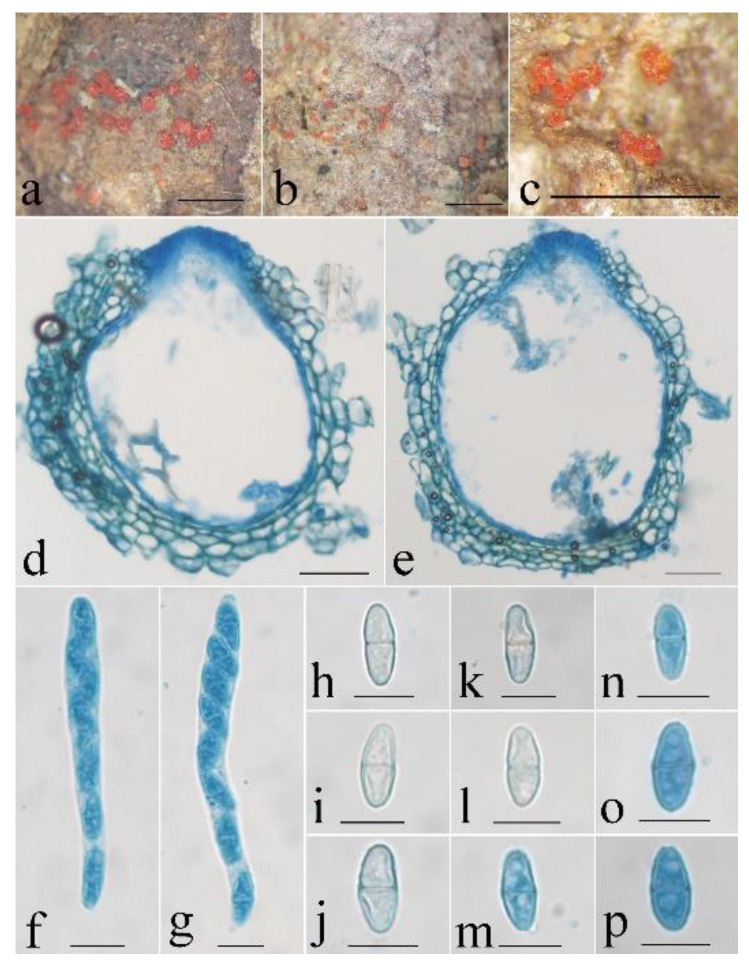
*Neocosmospora maoershanica* (HMAS 247875). (**a**–**c**) Perithecia on twigs. (**d**,**e**) Longitudinal section through perithecium. (**f**,**g**) Ascus with ascospores. (**h**–**p**) Ascospore. Scale bars: (**a**–**c**) = 1 mm, (**d**,**e**) = 50 μm, (**f**–**p**) = 10 μm.

**Figure 9:**
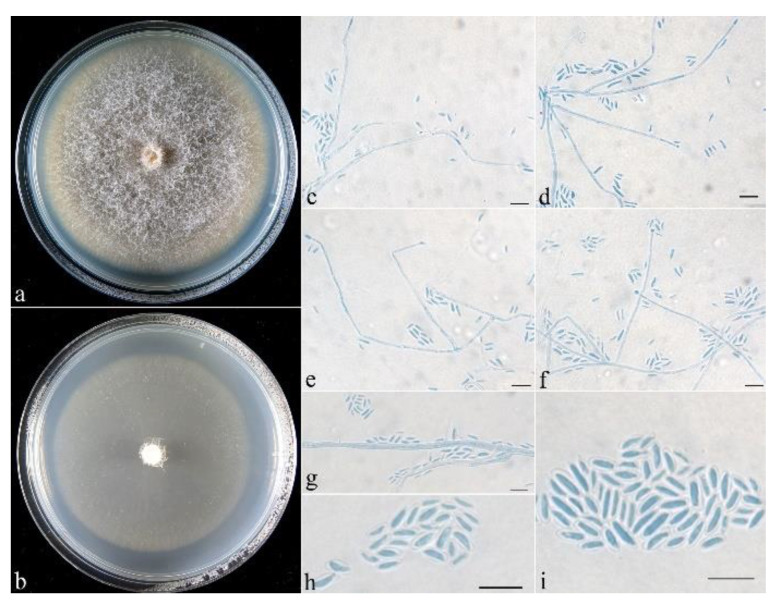
*Neocosmospora maoershanica* (CGMCC 3.24870). (**a**,**b**) Colonies after 1 wk at 25 °C ((**a**) on PDA, (**b**) on SNA). (**c**–**g**) Conidiophores and microconidia. (**h**,**i**) Microconidia. Scale bars: (**c**–**h**) = 10 μm.

***Neocosmospora maoershanica*** Z.Q. Zeng & W.Y. Zhuang, sp. nov. Figure 8 and Figure 9.

Fungal Names: FN 571311.

Etymology: The specific epithet refers to the type locality.

Typification: CHINA. Guangxi Zhuang Autonomous Region, Guilin, Maoershan, on twigs, 7 December Oct 2019, Z.Q. Zeng, & H.D. Zheng 12500 (holotype HMAS 247875, ex-type culture CGMCC 3.24870).

Mycelium was visible neither around ascomata nor on natural substrates. The characteristics of the ascomata were as follows: perithecial, superficial, solitary to gregarious, non-stromatic, subglobose to globose, or pyriform, with or without an inconspicuous papilla; orange-red when fresh, red when dry, turning dark red in 3% KOH, becoming yellow in 100% LA; and 216–294 × 176–255 μm. The perithecial surface was slightly roughened. The perithecial wall of two layers was 15–55 µm thick; the outer layer of textura angularis was 10–43 μm thick, the cells were 5–10 × 4–8 μm, the walls were 1–1.2 μm thick; the inner layer of textura prismatica was 5–12 μm thick, the cells were 5–12 × 2–3 μm, the walls were 0.8–1 μm thick. Asci were cylindrical to cylindrical-clavate, with a round and simple apex, (6–)8-spored and 55–85 × 5–8 µm. Ascospores were ellipsoidal to oblong, (0–)1-septate, hyaline to light brown, smooth-walled, uniseriate and overlapping obliquely, and 9–16 × 4.5–8 μm.

On PDA, the colony was 80 mm in diam. after 7 d at 25 °C, and the surface was cottony, with a dense, whitish aerial mycelium forming pale violet pigments. On SNA, the colony was 68 mm in diam. after 7 d at 25 °C, and the surface was floccose, with a sparse, whitish aerial mycelium. Conidiophores were acremonium- to verticillium-like, and the phialides were subulate, subcylindrical, acerose, hyaline, smooth-walled, 14–75 μm long, and 1.2–1.6 μm wide at the base. Microconidia were ellipsoidal, rod-shaped, bullet-shaped, hyaline, smooth-walled, 0(–1)-septate, and 3–13 × 2–4 μm. Macroconidia and chlamydospores were not observed.

Notes: Morphologically, *N. maoershanica* most resembles *N. oblonga* Sand.-Den. & Crous in having verticillium-like conidiophores and ellipsoidal to bullet-shaped, 0(–1)-septate, hyaline, and smooth-walled microconidia and lacking macroconidia [10]. However, *N. oblonga* differs in having a white to pale straw colony on PDA, longer microconidia (5–22 μm long), and the production of globose to subglobose chlamydospores. Sequence comparisons revealed that there are 13 bp and 23 bp differences detected for the CAM and RPB2 regions, respectively. Phylogenetically, *N. maoershanica* is closely associated with *N. longissima* (BIBP = 0.99) (Figure 1). The latter differs in forming an umber to rust colony on PDA and producing wedge-shaped macroconidia and abundant globose to obpyriform chlamydospores [10].

## 4. Discussion

Since the monotypic genus *Neocosmospora* was established [42], many species were described subsequently [43,44,45,46]. Later, the genus *Haematonectria* Samuels & Nirenberg, typified by *H. haematococca* (Berk. & Broome) Samuels & Rossman, was introduced by Rossman et al. [1] who stated that its ascomatal morphology and asexual features clearly distinguished it from *Neocosmospora*. Along with the information accumulated in phylogenetic studies, it has been indicated that the two genera are congeneric [40,47], and *Neocosmospora* was recommended as the preferable name according to the priority concept [2]. The taxonomic opinion became widely accepted [3,5,6,9,10,18], including the present research.

The phylogenetic tree based on combined analyses of CAM, ITS, RPB2, and TEF1 sequences showed that the five *Neocosmospora* strains (CGMCC 3.24866, 3.24867, 3.24868, 3.24869, and 3.24870) grouped with the representative species of the genus, which verified their taxonomic placements (Figure 1). *Neocosmospora dimorpha* (CGMCC 3.24867) clustered with *N. anhuiensis* (CGMCC 3.24869) (BIPP/MLBP = 1.0/99%), and they further grouped with *N. silvicola* (CBS 123846) (BIPP/MLBP = 0.99/78%). However, *N. silvicola* can be easily distinguished from *N. dimorpha* by its scarlet and ochreous to citrine pigment on PDA and globose to subglobose chlamydospores [10]. *Neocosmospora maoershanica* (CGMCC 3.24870) is related to *N. longissima* (CBS 126407) (BIBP = 0.9) but differs by its pale violet pigments produced on PDA and the lack of macroconidia and chlamydospores in culture [10]. *Neocosmospora aurantia* (CGMCC 3.24866) is phylogenetically related with, but clearly separated from, *N. phaseoli* (CBS 26550) (BIPP/MLBP = 1.0/98%), and the latter is distinguished by its shorter macroconidia, larger microconidia, and the production of subglobose to ellipsoidal chlamydospores [41]. *Neocosmospora galbana* (CGMCC 3.24868) grouped with *N. lithocarpi* (LC 1113) (BIPP/MLBP = 1.0/93%) and differs by its faster growth rate and greenish-yellow colony on PDA, narrower macroconidia, smaller microconidia without septa, and the lack of chlamydospores [9].

Since *N. sphaerospora* (Q.T. Chen & X.H. Fu) Sand.-Den. & Crous (as *Fusarium sphaerosporum* Q.T. Chen & X.H. Fu) and *N. petroliphila* (Q.T. Chen & X.H. Fu) Sand.-Den. & Crous (as *Fusarium solani* var. *petroliphilum* Q.T. Chen & X.H. Fu) were first reported by Chen et al. [48], five additional new species of the genus were successively described from different provinces of China [3,9,49,50]. Among these seven species, four taxa, namely, *N. lithocarpi*, *N. pallidimors* Tibpromma, Karun., Karasaki & P.E. Mortimer, *N. petroliphila*, and *N. sphaerospora* are only known with their asexual stages [3,9,48]. In the present study, five holomorphic novel taxa of the genus were introduced, which significantly increase the species diversity of the genus in the country. Further surveys in the unexplored regions will update our understanding of the species diversity of nectrioid fungi in China and the world [51]. Combining their sexual stages with their asexual stages will give a better comprehension of the whole fungus.

## Figures and Tables

**Table 1:** Sequences of *Neocosmospora* species used in this study.

Species	Strain/Herbarium Numbers	GenBank Accession Numbers			
		CAM	ITS	RPB2	TEF1
*Geejayessia atrofusca*	NRRL 22316	-	AF178423	JX171609	AF178361
*Geejayessia cicatricum*	CBS 125552	-	MH863560	HQ728153	HM626644
*Neocosmospora acutispora*	NRRL 22574	MW834122	NR169884	EU329514	AF178345
*Neocosmospora ambrosia*	NRRL 22346	-	EU329669	EU329503	FJ240350
*Neocosmospora anhuiensis*	CGMCC 3.24869	**OR014310 ^a^**	**OQ842733**	**OQ866525**	**OQ866530**
*Neocosmospora aurantia*	CGMCC 3.24866	**OR014308**	**OQ842731**	**OQ866523**	**OQ866528**
*Neocosmospora caricae*	ES216M	-	OK422518	OK415859	OK539518
*Neocosmospora cryptoseptata*	NRRL 22412	MW834126	NR172368	EU329510	AF178351
*Neocosmospora cyanescens*	CBS 51882	MW218064	AB190389	LR583826	LR583605
*Neocosmospora dimorpha*	CGMCC 3.24867	**OR014309**	**OQ842732**	**OQ866524**	**OQ866529**
*Neocosmospora euwallaceae*	NRRL 54722	KU171422	JQ038014	JQ038028	JQ038007
*Neocosmospora ferruginea*	NRRL 32437	MW834132	DQ094446	EU329581	DQ246979
*Neocosmospora galbana*	CGMCC 3.24868	**OR014307**	**OQ842730**	**OQ866527**	**OQ866532**
*Neocosmospora keleraja*	CBS 125720	MW834138	LR583720	LR583834	LR583612
*Neocosmospora kuroshio*	CBS 142642	MW834140	LR583723	LR583837	KX262216
*Neocosmospora kurunegalensis*	CBS 119599	MW834141	JF433036	LR583838	DQ247511
*Neocosmospora lithocarpi*	LC 1113	-	MW016711	MW474697	MW620172
*Neocosmospora longissima*	CBS 126407	MW834144	NR178144	LR583846	LR583621
*Neocosmospora macrospora*	CPC 28191	MW218078	NR163291	LT746331	LT746218
*Neocosmospora mahasenii*	CBS 119594	MW834145	JF433045	LT960563	DQ247513
*Neocosmospora maoershanica*	CGMCC 3.24870	**OR014311**	**OQ842734**	**OQ866526**	**OQ866531**
*Neocosmospora mori*	NRRL 22230	MW834149	AF178420	EU329499	AF178358
*Neocosmospora nelsonii*	CBS 30975	MW834152	MW827630	MW847904	MW847907
*Neocosmospora nirenbergiana*	NRRL 22387	MW834153	NR169883	EU329505	AF178339
*Neocosmospora oblonga*	CBS 130325	MW834154	LR583746	LR583853	LR583631
*Neocosmospora oligoseptata*	NRRL 62579	MW834155	KC691566	LR583854	KC691538
*Neocosmospora phaseoli*	CBS 26550	KM231380	MH856617	KM232375	HE647964
*Neocosmospora pisi*	CBS 123669	MW834159	KM231796	KM232364	KM231925
*Neocosmospora pseudensiformis*	CBS 130.78	MW834162	LR583759	LR583868	DQ247635
*Neocosmospora quercicola*	CBS 14190	MW834164	NR178125	LR583869	DQ247634
*Neocosmospora regularis*	CBS 23034	MW834168	LR583763	LR583873	LR583643
*Neocosmospora rekana*	CMW 52862	-	MN249094	MN249137	MN249151
*Neocosmospora robusta*	NRRL 22395	MW834169	NR172367	EU329507	AF178341
*Neocosmospora samuelsii*	CBS 114067	MW834170	NR178127	LR583874	LR583644
*Neocosmospora silvicola*	CBS 123846	MW834172	LR583766	LR583876	LR583646
*Neocosmospora spathulata*	NRRL 28541	MW218091	EU329674	EU329542	DQ246882
*Neocosmospora vasinfecta*	CBS 446.93	MW834175	LR583791	LR583898	LR583670

^a^ Numbers in bold indicate the newly generated sequences.

## Data Availability

The names of the new species were formally registered in the database Fungal Names (http://www.fungalinfo.net/fungalname/fungalname.html (accessed on 20 February 2023)). Specimens were deposited in the Herbarium Mycologicum Academiae Sini-cae (https://nmdc.cn/fungarium/ (accessed on 18 February 2023)). The newly created sequences were deposited in GenBank (https://www.ncbi.nlm.nih.gov/genbank (accessed on 20 May 2023)).
